# Effects of wearable ankle robotics for stair and over-ground training on sub-acute stroke: a randomized controlled trial

**DOI:** 10.1186/s12984-021-00814-6

**Published:** 2021-01-29

**Authors:** Ling-Fung Yeung, Cathy C. Y. Lau, Charles W. K. Lai, Yannie O. Y. Soo, Man-Lok Chan, Raymond K. Y. Tong

**Affiliations:** 1 Department of Biomedical Engineering, The Chinese University of Hong Kong, Shatin, Hong Kong; 2 Physiotherapy Department, Shatin Hospital, Ma On Shan, Hong Kong; 3 Department of Medicine and Therapeutics, Prince of Wales Hospital, Shatin, Hong Kong; 4 Physiotherapy Department, Tung Wah Hospital, Sheung Wan, Hong Kong

**Keywords:** Randomized controlled trial, Stroke rehabilitation, Gait training, Robotics, Ankle–foot orthosis, Stair ambulation

## Abstract

**Background:**

Wearable ankle robotics could potentially facilitate intensive repetitive task-specific gait training on stair environment for stroke rehabilitation. A lightweight (0.5 kg) and portable exoskeleton ankle robot was designed to facilitate over-ground and stair training either providing active assistance to move paretic ankle augmenting residual motor function (power-assisted ankle robot, PAAR), or passively support dropped foot by lock/release ankle joint for foot clearance in swing phase (swing-controlled ankle robot, SCAR). In this two-center randomized controlled trial, we hypothesized that conventional training integrated with robot-assisted gait training using either PAAR or SCAR in stair environment are more effective to enhance gait recovery and promote independency in early stroke, than conventional training alone.

**Methods:**

Sub-acute stroke survivors (within 2 months after stroke onset) received conventional training integrated with 20-session robot-assisted training (at least twice weekly, 30-min per session) on over-ground and stair environments, wearing PAAR (n = 14) or SCAR (n = 16), as compared to control group receiving conventional training only (CT, n = 17). Clinical assessments were performed before and after the 20-session intervention, including functional ambulatory category as primary outcome measure, along with Berg balance scale and timed 10-m walk test.

**Results:**

After the 20-session interventions, all three groups showed statistically significant and clinically meaningful within-group functional improvement in all outcome measures (p < 0.005). Between-group comparison showed SCAR had greater improvement in functional ambulatory category (mean difference + 0.6, medium effect size 0.610) with more than 56% independent walkers after training, as compared to only 29% for CT. Analysis of covariance results showed PAAR had greater improvement in walking speed than SCAR (mean difference + 0.15 m/s, large effect size 0.752), which was in line with the higher cadence and speed when wearing the robot during the 20-session robot-assisted training over-ground and on stairs.

**Conclusions:**

Robot-assisted stair training would lead to greater functional improvement in gait independency and walking speed than conventional training in usual care. The active powered ankle assistance might facilitate users to walk more and faster with their paretic leg during stair and over-ground walking.

*Trial registration:* ClinicalTrials.gov NCT03184259. Registered on 12 June 2017.

## Introduction

Stroke is a leading cause of long-term disability [1]. Person with stroke commonly suffer from foot drop problem, with high falling risk because the affected foot would drag on the ground and easily stumble on obstacles [2]. Effective gait recovery is essential to improve quality of life [3] and independency of stroke survivors [4]. Early rehabilitation in sub-acute phase is known to be more effective [4, 5], but after completion of conventional gait rehabilitation, approximately 50–60% of stroke survivors still experienced some degree of motor impairment, and about half of them were still at least partly dependent in walking [6].

Conventional gait rehabilitation often involved intensive, repetitive, and task-specific gait practices [7–9], mainly walking on level surfaces; while some studies showed intensive stepping training on stairs could improve walking speed and balance in sub-acute [10] and chronic stroke [11, 12]. Previous researches showed sub-acute stroke survivors participating in early gait training together with electromechanical-assisted robotics, such as Lokomat, Gait Trainer, and G-EO system, could improve recovery of gait independency to a certain extent [4, 9]. But these gait training systems confined the users in constant treadmill-like setting. An important goal of gait rehabilitation is to enable independent walking and confidence to enhance their quality of life as soon as possible without assistance from caregivers [4, 13], and the real-world walking environment would involve varying terrains, like obstacles, slopes, and stairs. Following a task-specific training approach [8], early stroke rehabilitation on stair negotiation could potentially enhance gait re-education for better outcome on gait independency at hospital discharge. However, intensive stair training was not a common clinical practice for sub-acute stroke because of safety concern on stair environment.

To facilitate stair negotiation of person with stroke, ankle–foot orthoses (AFO) were commonly prescribed to passively support the dropped foot during swing phase [14, 15]. A meta-analysis showed the immediate effect of applying AFO could significantly improve walking speed and balance [16], but long-term application of rigid AFO did not influence gait pattern of sub-acute stroke, with limited therapeutic effects [17]. Studies also showed conventional rigid AFO did not mimic normal ankle movement during walking and might impose undesirable restrictions on ankle range of motion [14, 18]. In particular, restricted ankle joint could raise safety issue when the user was negotiating stairs onto a lower level. The major challenge was how to position the dropped foot properly and consistently to avoid tripping onto the step edge when negotiating stairs. In these cases, wearable ankle robotics could be a viable solution to enable better ankle joint control during stair training.

Existing lower-limb rehabilitation robots were often limited by their device weight and portability for stair environment, so few of these devices could be evaluated and developed to the stage of commercialization and clinical application [19]. G-EO system was a commercialized end-effector robot that could simulate stair climbing in a treadmill-like environment by moving foot plates in cycle to reproduce step length and height of stairs, but the system was bulky and stationary. The randomized controlled trial (RCT) evaluated the stair version of G-EO system focused on balance training of chronic stroke subjects [20]. Portable-power ankle–foot orthosis developed in the University of Illinois used a pneumatic bidirectional rotatory actuator to provide untethered ankle assistance on level ground and stairs. The robot and control algorithm were evaluated on healthy subjects (n = 5) as a technical feasibility test [21]. Recent development of ReStore exo-suit (ReWalk Robotics, USA) featured a soft garment-like design driven by Bowden cable, could offer potential solution to reduce device weight and bulkiness of robot at ankle joint [22]. Similar ankle rehabilitation robotics have also shown their potential to be an alternative gait rehabilitation for stroke [19, 23–26], like Anklebot [8]. However, few studies investigated how impaired subjects would respond to these rehabilitation robots immediately during walking on stairs, and few studies reported the therapeutic effects of these devices in multi-center RCT setting [4, 19, 25, 26].

Our research team has already developed an exoskeleton ankle robot for gait training on people with chronic stroke and foot drop problem [27]. The robot could provide active assistive torque to facilitate paretic ankle dorsiflexion for stair clearance and assist plantarflexion for loading response (power-assisted mode); alternatively, the motor could also lock the ankle joint at neutral position for foot clearance during swing phase like a rigid AFO, and release the lock for free ankle movement in stance phase (swing-controlled mode). The lightweight (0.5 kg on the paretic ankle) and portable design of this robot-assisted AFO could make a potential rehabilitation tool for gait training of hemiplegic stroke on over-ground walking and stair ascending/descending.

Our previous study has already evaluated the ankle robot in a pilot RCT for chronic stroke, which showed significant improvement in gait independency and enhanced gait confidence at heel strike after wearing this robot for 20-session stair and over-ground gait training [28]. Nevertheless, early rehabilitation in sub-acute stroke is known to have greater impact on functional recovery than chronic stroke. The primary objective of the current study was to evaluate the effects on sub-acute stroke survivors with the wearable robot-assisted AFO. We hypothesized conventional training integrated with 20-session robot-assisted training (10-min stair training plus 20-min over-ground walking) in early stroke wearing the ankle robot either in power-assisted mode or the swing-controlled mode, would result in greater functional improvement in gait independency, balance, and speed, than conventional training only. We further hypothesized active powered assistance would have more benefit on functional improvement than passive ankle swing control during the robot-assisted training.

## Methods

### Subjects

This was a two-center RCT conducted in Hong Kong between 2017 and 2019. Sub-acute stroke survivors were screened and recruited from two local hospitals: Hospital S and Hospital T. This study was approved by the Institutional Review Board of the hospitals and was designed following the principle of the Declaration of Helsinki. All recruited subjects read and signed consent form before participation.

Recruited subjects satisfied the following inclusion criteria, including (1) first episode of stroke within 2 months, (2) hemiparesis resulting from unilateral ischemic or hemorrhagic stroke, (3) ability to walk with one-person assistance (functional ambulatory category, FAC ≥ 1), and (4) sufficient cognition to follow instructions and understand the content and purpose of the study. Subjects were excluded if he/she had (1) uncontrolled cardiovascular or respiratory disorders, (2) moderate to severe contractures in lower extremities (modified Ashworth scale, MAS > 2 at ankle, knee, or hip), or (3) orthopedic or muscle disorders that affected mobility.

### Intervention

Recruited subjects were randomly allocated into three groups by drawing lots: (1) power-assisted ankle robot (PAAR), (2) swing-controlled ankle robot (SCAR), and (3) conventional training (CT). All subjects received conventional rehabilitation protocol (physiotherapy and occupational therapy) prescribed by rehabilitation team of the inpatient training centers for 2 h/weekday, including standard lower-limb exercises on standing, balance, stepping, and walking.

For subjects who were assigned in PAAR and SCAR, 30-min robot-assisted training (at least two sessions/week, total 20 sessions) were integrated into their conventional training routine (2 h/weekday) without time compensation. Each robot-assisted training session consisted of 10-min over-ground walking, followed by 10-min stair training (ascending/descending), then another 10-min over-ground walking. The two training centers had similar settings: having staircase with handrail (5–10 steps with 120–150 mm step height, 1.2–1.5 m width, 350–400 mm depth) and long corridor (≥ 10 m) cleared of obstacle with minimal turning. Subjects were free to take break anytime but resting was also counted in the training time. The whole session lasted around 45 min including robot setup (don/doff) time. A trainer walked beside the affected side of the subject and held subject’s waist belt all the time to ensure safety. The trainer administered verbal cue on head/trunk extension in case of increased trunk kyphosis, or mid-line awareness when subjects leaned on the unaffected side. Subjects used their own walking aids prescribed by the hospital rehabilitation team, including walking cane, quadruped stick, and walker. The rehabilitation team checked the subject’s vital sign and reviewed his/her functional capability before each session. The trainer regularly asked the subjects if they felt any pain and discomfort during training. The number of stairs and walking distance covered were documented in each session as a record of training intensity and capacity.

### Exoskeleton ankle robot

**Fig. 1:**
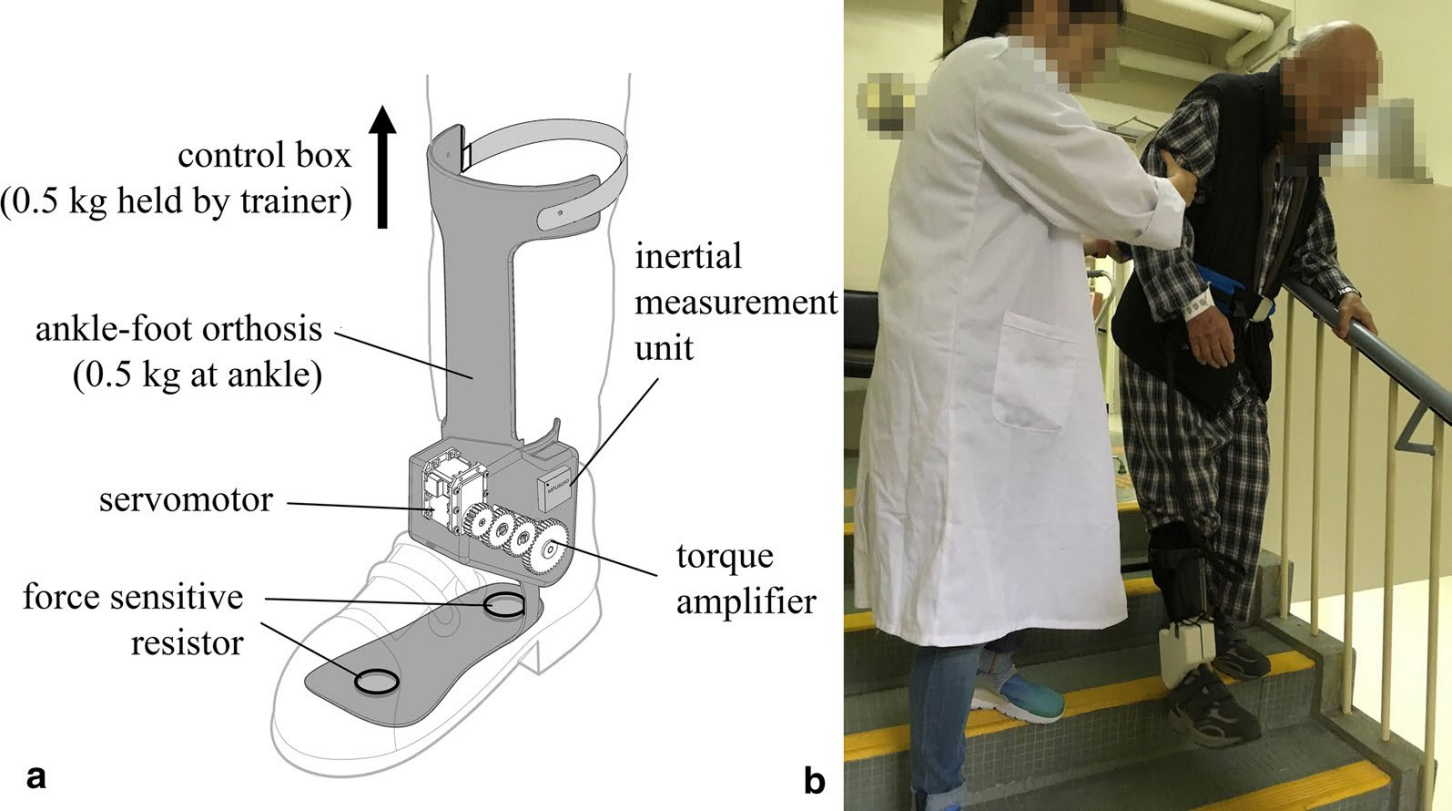
**a** Exoskeleton ankle robot used in this study for robot-assisted stair training of sub-acute stroke survivors in both power-assisted ankle robot (PAAR) group and swing-controlled ankle robot (SCAR) group. **b** Robot-assisted stair training

Both PAAR and SCAR were provided with the same exoskeleton ankle robot but in different operation mode adjustable by the trainer. The robot was worn inside subject’s footwear on the affected side throughout each robot-assisted training session (Fig. 1). The wearable robot was modified from an articulated AFO with the ankle joint coupled with a rotatory servomotor (Dynamixel MX-106R, ROBOTIS, South Korea) and a torque amplifier (1:1.67 gear ratio) that can provide powered assistance in ankle dorsiflexion/plantarflexion directions. The robot can identify changes in foot loading and gait phases using embedded force sensitive resistors (FSR-402, Interlink Electronics, USA) placed under heel and forefoot. An inertial measurement unit (MPU6050, 6-axis MotionTracking, InvenSense, USA) mounted on the shank can measure leg tilting angle for classifying user walking intention on level and stair walking [27]. The robot weighted 0.5 kg (including AFO and motor) on the leg, with the control box (0.5 kg) held by the trainer.

The ankle robot in PAAR mode was intended to provide powered ankle assistance together with residual motor function to facilitate over-ground walking and stair training. If the robot detected walking intention in either over-ground walking or stair ascending, the servomotor generated sufficient constant torque on the affected ankle in dorsiflexion direction to prevent foot drop and to facilitate foot clearance with around 10° ankle dorsiflexion throughout swing phase of walking, until heel strike was detected and then ankle joint was free to move in stance phase. Contrary, if the subject was stair descending, the servomotor generated constant torque in plantarflexion direction to facilitate loading response when the affected foot was landing on the lower step, then the ankle joint was free to move when the heel touched the floor. To calibrate dorsiflexion assistance, subjects were told to perform voluntary maximum ankle dorsiflexion on the dropped foot, while the motor torque gradually increased in dorsiflexion direction until the paretic ankle reached 10° dorsiflexion. To calibrate plantarflexion assistance, subjects were told to stand quietly on both leg while the motor torque increased gradually in plantarflexion direction until the torque was sufficient to uplift the heel to 10° plantarflexion on affected side. The calibration was performed by the trainer at the beginning of each session to adjust for any progression of functional changes throughout the 20-session gait training. The calibrated ankle torque requirement matched with previous research 3.6 ± 2.4 Nm on stroke subjects (n = 80) with mild spasticity (MAS ≤ 2) [18].

The ankle robot in SCAR mode acted as a swing-controlled orthosis, which switched between locked and unlocked ankle joint based on the gait phases [14]. Whenever the robot detected terminal stance as the foot was lifted up from the ground, the ankle joint was locked by the servomotor in the neutral position to prevent foot drop condition during swing phase for foot clearance [24], effectively acted as a rigid AFO. When heel strike and foot contact with the ground were detected, the servomotor released the ankle joint to allow unimpeded forward ankle rocker during stance phase. Similar passive swing-controlled AFO had been proposed by previous researches showing these devices were able to prevent foot drop and enhance gait stability [14].

### Outcome measures

Clinical assessments were carried out by blinded assessors within a week before the intervention (Pre) and within a week after the intervention (Post). The same assessor administered both Pre and Post assessment of a subject. All assessors were blinded to group allocation. Clinical assessments were selected based on a meta-analysis that aimed to evaluate the effectiveness of wearing AFO, which recommended outcome measures targeting on mobility, walking, and balance [16, 29]. All clinical scores were assessed on subjects without using any assistive devices, neither the ankle robot nor any orthosis subjects wore.

The primary outcome measure was FAC, which was used to classify gait independency based on a six-point scale, ranging from FAC = 0 “needs help from at least two persons to walk” to FAC = 5 “can walk independently anywhere, including uneven surfaces and stairs”. Previous study determined that FAC ≥ 4 could predict community ambulation at 6-month with 100% sensitivity and 78% specificity after 4-week rehabilitation [4, 13].

The secondary outcome measures included Berg balance scale (BBS) and timed 10-m walk test (10MWT). BBS was used to assess static and dynamic balance ability based on 14 functional tasks with varying difficulty, including sitting, standing, transfer, reaching, stepping, and turning. Each task was rated on a five-point scale, ranging from 0 to 4 based on the performance of the subject in completing the activity. The highest BBS score was 56, while the score of 45 had been shown to be a cut-off score for greater functional independency and lower fall risk for stroke survivors [29]. 10MWT measured the self-selected walking speed in meter per second over a short distance. The uses of walking aids and manual assistance were documented and made consistent for Pre and Post assessments. Studies indicated walking speed had good correlation with functional independency and disability level [13, 30]. Stroke subjects who walked with self-selected speed > 0.4 m/s were considered at least limited community ambulators [31].

### Statistical analysis

The power analysis for sample size calculation was based on our previous RCT that investigated the effects of robot-assisted gait training on functional independency of chronic stroke survivors [28], with the between-group difference in FAC score had effect size 0.471. The estimated sample size for the current study was 48 for three groups with 0.8 power (1-β) [32]. The power analysis was performed using G*Power version 3.1.9.6.

The statistical analysis aimed to evaluate any significant difference between robot-assisted trainings and conventional training on sub-acute stroke survivors. All outcome measures were analyzed based on the intention-to-treat principle, which used the last-observation-carried-forward method to impute the last available data to missing entries for any drop-out. Analysis of covariance (ANCOVA) was used to compare the improvement (Post) scores in FAC, BBS and 10MWT between groups. To reduce the expected confounding effect of the variation in baseline clinical scores, we adjusted the group means using baseline (Pre) scores as covariate. If ANCOVA revealed significant effects, post-hoc comparison between groups were tested using Mann–Whitney U-test for ordinal variables (FAC and BBS) and independent samples t-test for continuous scales (10MWT). To explore the practical significance of group differences, effect sizes were calculated as follows:

$$Effect Size = {{\left( {Mean_{Group1} - Mean_{Group2} } \right)} \mathord{\left/ {\vphantom {{\left( {Mean_{Group1} - Mean_{Group2} } \right)} {SD_{Pooled} }}} \right. \kern-\nulldelimiterspace} {SD_{Pooled} }}$$

The established criteria of the effect size, which reflects the treatment effect within the target population, were small (< 0.41), medium (0.41 to 0.70), or large (> 0.70) [33]. Statistical results were reported with the effect size in 95% confidence interval (95%CI). Two-tailed level of significance set at 5%. Statistical analysis was performed using IBM SPSS Statistics Version 23 (IBM Corp., USA).

## Results

**Fig. 2:**
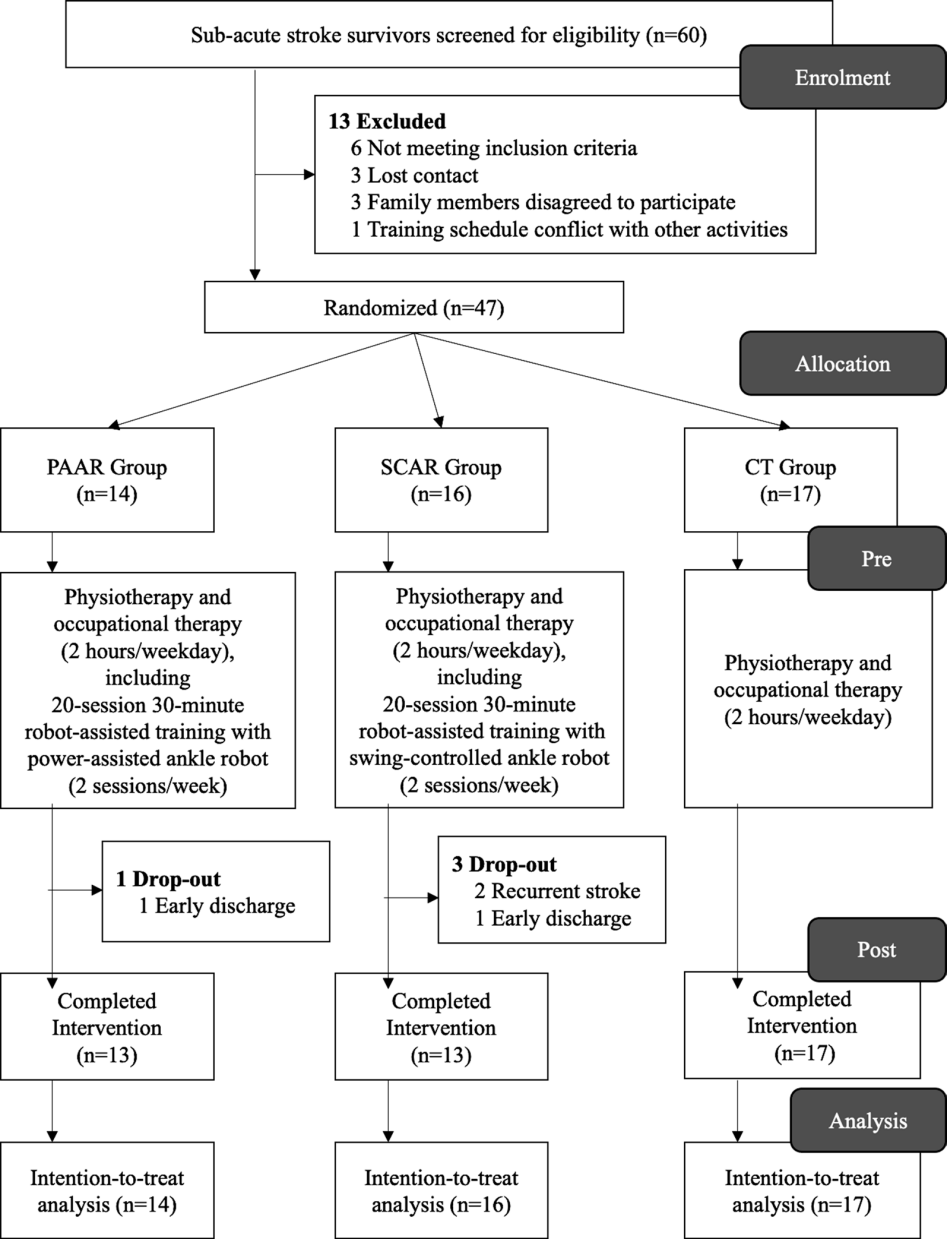
CONSORT participant flow chart.

Total 60 sub-acute stroke survivors from two participating hospitals were screened for eligibility from July 2017 to December 2018. Stroke survivors who met eligibility criteria were randomized and allocated into PAAR (n = 14), SCAR (n = 16), and CT (n = 17) (Fig. 2). If subjects in PAAR and SCAR were discharged from the hospital before completing the 20-session robot-assisted training, they were invited to continue the remaining sessions in out-patient day-care rehabilitation center of the same hospital facility. Total four stroke survivors had not completed the 20-session (1 PAAR and 3 SCAR) because of recurrent stroke or difficulties to attend day-care facility after early discharge. All drop-out subjects had finished at least ten sessions before discharge. No serious adverse event or important harm was reported. Subjects did not report any discomfort after our fitting adjustment and adding soft padding.

Apart from stroke type of all SCAR subjects were ischemic (χ(1) = 6.70, p = 0.035), no statistically significant between-group difference was found in baseline clinical assessment scores and demographic characteristics (Table 1). No significant difference between two training centers.

**Table 1:** Demographic characteristics

Characteristics	All subjects (n = 47)	PAAR (n = 14)	SCAR (n = 16)	CT (n = 17)
Age (years)^a^	65.5 ± 9.6	64.6 ± 12.6	68.3 ± 10.3	63.6 ± 5.2
Gender (male/female)	24/23	8/6	8/8	8/9
Affected limb (left/right)	23/24	9/5	8/8	6/11
Stroke type (ischemic/hemorrhagic)	38/9	11/3	16/0*	11/6
Stroke duration before screening (days)^a^	27 ± 17	23 ± 14	29 ± 15	28 ± 21
Training duration (days)^a^	38 ± 22	35 ± 10	45 ± 28	33 ± 22

^a^Values present in mean ± SD

*p < 0.05, significant within-group difference

**Fig. 3:**
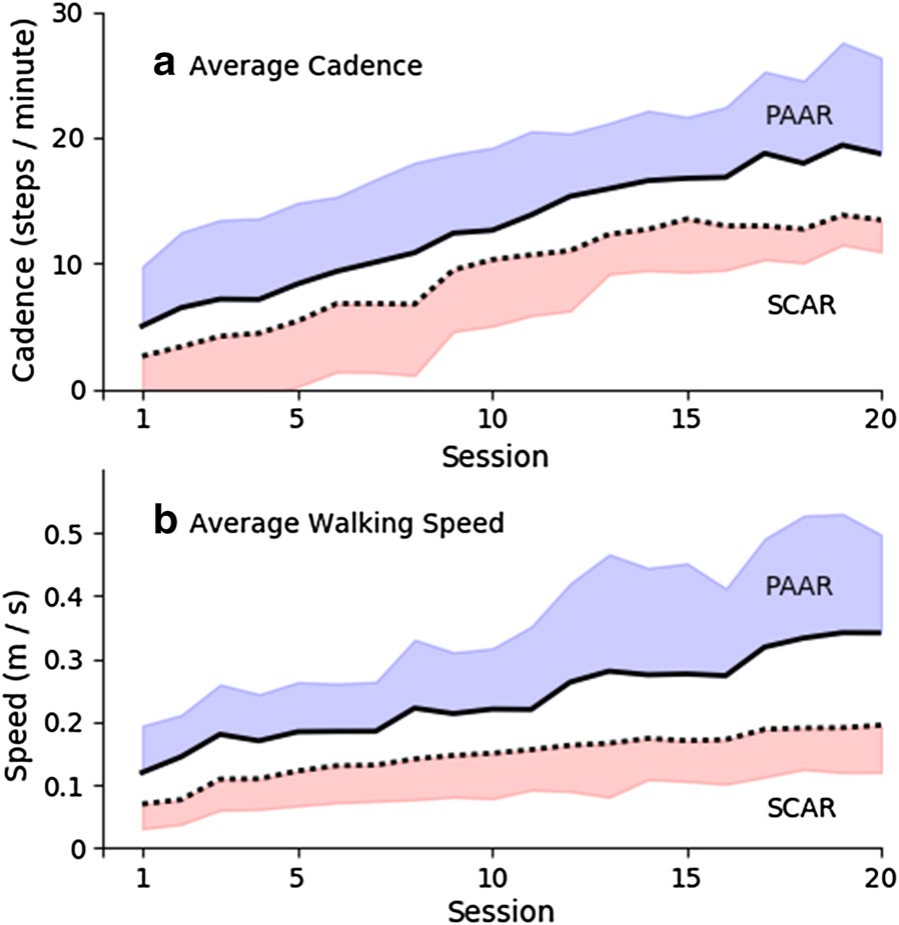
Training intensity across the 20-session robot-assisted training for power-assisted ankle robot (PAAR, solid line) and swing-controlled ankle robot (SCAR, dotted line), in terms of **a** average cadence during 10-min stair walking, and **b** average walking speed during 20-min over-ground walking. The shaded area represents the SD

Results revealed when wearing the robot during robot-assisted training, more active power assistance from PAAR could facilitate subjects to walk higher cadence and speed during stair climbing and over-ground walking than SCAR (see Table 2 and Fig. 3). Both groups showed significantly increased cadence and speed across 20-sessions training (p < 0.001), but PAAR subjects covered significantly more number of stairs (+ 6 steps/min at the 20th session, 95% CI [+ 0.0, + 1.2], t = 2.085, p = 0.049) and walked significantly faster speed (+ 0.15 m/s at the 20th session, 95% CI [+ 0.04, + 0.25], t = 2.837, p = 0.009) than SCAR subjects.

**Table 2:** Outcome measures of clinical scores at baseline (Pre) present in mean ± SD, and within-group differences after gait training (Post–Pre) present in mean difference (95%CI)

	PAAR (n = 14)	SCAR(n = 16)	CT (n = 17)
FAC (max. 5)			
Pre	1.9 ± 0.7	2.2 ± 0.8	2.2 ± 1.0
Post–pre	+ 1.4 (+ 1.0, + 1.9)***	+ 1.4 (+ 0.9, + 2.0)***	+ 0.9 (+ 0.4, + 1.3)**
% Independent walker (FAC ≥ 4)	57.1%	56.3%	29.4%
BBS (max. 56)			
Pre	24.0 ± 11.3	30.7 ± 14.6	25.9 ± 14.4
Post–pre	+ 18.8 (+ 13.1, + 24.4)***	+ 12.6 (+ 6.2, + 18.9)**	+ 14.4 (+ 9.4, + 19.3)***
% Post–pre > MCID	71.4%	50.0%	47.1%
10MWT (m/s)			
Pre	0.13 ± 0.16	0.14 ± 0.16	0.14 ± 0.18
Post–pre	+ 0.32 (+ 0.18, + 0.46)***	+ 0.17 (+ 0.09, + 0.25)**	+ 0.17 (+ 0.06, + 0.29)**
% Post–pre > MCID	71.4%	56.3%	41.2%
Stair active training time (s)^a^			
Baseline	464 ± 100	238 ± 67	
10th-session	476 ± 97	401 ± 145*	
20th-session	480 ± 121	427 ± 154*	
Number of stairs covered (step)			
Baseline	72 ± 18	40 ± 26	
10th-session	115 ± 36**	83 ± 49	
20th-session	154 ± 85**	95 ± 40*	
Walk active training time (s)^†^			
Baseline	874 ± 212	806 ± 173	
10th-session	1010 ± 138*	1044 ± 162**	
20th-session	1072 ± 171**	1070 ± 148***	
Walking distance covered (m)			
Baseline	118.8 ± 70.5	64.3 ± 31.9	
10th-session	225.7 ± 161.9***	168.7 ± 90.7***	
20th-session	362.5 ± 189.1***	212.0 ± 100.7***	
Assistive torque level (Nm)			
Baseline	3.6 ± 0.8		
10th-session	3.6 ± 0.7		
20th-session	3.5 ± 0.6		

For robot-assisted training, the assistive torque level, and the training intensity in terms of active training time and stairs/distance covered at three time points (baseline, 10th-, and 20th-session) present in mean ± SD

*p < 0.05, **p < 0.01, ***p < 0.001, significant within-group difference

^†^Active training time = assigned training (600 s for stair, 1200 s for walk) minus resting time

Table 2 showed the changes in clinical scores of FAC, BBS, and 10 MWT before and after the interventions. There were significant within-group differences in all clinical scores (p < 0.005), indicating all three groups had functional improvements after the interventions.

Table 3 showed the adjusted between-group difference in clinical score improvement using baseline (Pre) as covariates. ANCOVA results showed subjects in SCAR had significantly better FAC improvement than CT; and PAAR had significantly greater improvement in walking speed than both SCAR and CT. BBS balance performance did not show significant difference between three groups.

**Table 3:** Adjusted between-group differences of clinical score improvement (Post–Pre) using baseline (Pre) as covariates

Outcome Measures	PAAR vs CT	SCAR vs CT	PAAR vs SCAR
FAC	+ 0.4 (− 0.2, + 1.0) [0.671]	+ 0.6 (+ 0.0, + 1.1)*[0.610]	− 0.2 (− 0.8, + 0.4) [0.010]
BBS	+ 3.6 (− 2.7, + 9.9) [0.458]	+ 0.3 (− 5.8, + 6.5) [0.166]	+ 3.2 (− 3.3, + 9.7) [0.567]
10MWT (m/s)	+ 0.15 (+ 0.0, + 0.29)*[0.641]	+ 0.0 (− 0.15, + 0.14) [0.020]	+ 0.15(+ 0.0, + 0.30)*[0.752]

All analysis of covariance between-group differences present in mean difference (95%CI) [Cohen's d index of effect size]

*p < 0.05, significant between-group difference

## Discussion

This study was one of the first clinical trials that applied robot assistance in stair training for sub-acute stroke. This RCT showed after 20-session, PAAR, SCAR, and CT had significant within-group functional improvements in gait independency (FAC), balance (BBS), and walking speed (10MWT). Between-group comparison suggested robot-assisted training (PAAR and SCAR) could result in significantly greater improvement in functional independency than conventional training in usual care (CT). In particular, powered assistance in PAAR that actively moved the paretic ankle to facilitate subjects were able to walk faster with higher cadence in the 20-session robot-assisted training when compared with the ankle-locking swing-controlled robot in SCAR. Our results demonstrated feasibility of intensive stair training using ankle robotics for stroke rehabilitation. More similar researches should be done in the future to confirm the value of intensive stair training in clinical application.

Our previous RCT on chronic stroke (n = 19) had compared PAAR with SCAR in similar experiment setting, which showed robot-assisted trainings were effective in chronic stroke, with FAC improved + 0.6 and walking speed + 0.07 m/s after 20-session training [28]. In the current study for sub-acute stroke, both PAAR and SCAR had + 1.4 improvement in FAC, with more than 56% of subjects turned from dependent walker (FAC < 4) at baseline to become independent walker (FAC ≥ 4) after intervention; while CT only had 29%. For walking speed, PAAR in the current study had + 0.32 m/s improvement, the greater proportion of sub-acute stroke subject walked faster than the minimal clinically important difference (MCID = 0.16 m/s), in PAAR (71.4%) vs CT (41.2%) (χ(1) = 5.290, p = 0.021) was in line with their improved gait independency [30]. These results agreed with several systematic reviews that supplementing conventional physiotherapy with electromechanical-assisted gait training in sub-acute stage would have greater functional improvement than chronic stage [4, 5, 23].

In contrary to existing clinical application of high-intensity task-specific gait training that often performed on treadmill or level ground [10, 34, 35], the current study demonstrated wearable robot-assisted training could even be implemented in simple stair environment as a feasible rehabilitation approach. Previous studies showed mild stair training in chronic stroke could improve physical activity level [11], trunk stability and balance [10, 12], walking speed and endurance [10, 36]. The robot-assisted stair training described in the current study required only one skilled trainer walking alongside the stroke subjects for safety and verbal cueing, while the posture adjustment for foot drop correction could be handled automatically by the robot itself [37]. Rehabilitation robotics are capable of delivering intensive, repetitive and adjustable gait assistance patterns while sharing workload of therapists [25, 37].

Effect sizes were computed for the three outcome measures (FAC, BBS, and 10MWT) to determine the strength of association for the statistically significant interactions (Table 3). Between-group comparison of FAC revealed a medium effect size between robot-assisted training and conventional training (PAAR vs CT 0.671, SCAR vs CT 0.610). This suggested a larger sample size would have possibly produced more statistically significant effect. The effect size difference between PAAR and SCAR were small in FAC (0.010), but the 4-week intervention showed a large effect size difference in 10MWT (0.752) and medium effect size difference in BBS (0.567). Hence, PAAR might be more favorable than SCAR toward functional improvement in walking speed and balance.

Comparison between PAAR and SCAR revealed an interesting finding about the effect of active powered assistance. During the 20-session robot-assisted training, PAAR could walk faster speed and higher cadence than SCAR when wearing the robot (Fig. 3), which implies PAAR that offered more active assistance to facilitate the ankle joint in dorsiflexion might be superior than SCAR that provided passive support to dropped foot for better foot clearance. These enhanced gait stabilities and walking speed in PAAR could be maintained even after removing the robotic assistance, as supported by the therapeutic effects in the Post clinical assessment scores. Hence, active robotic assistance might play an important role in the gait relearning; passive support, as in the SCAR device, offered relatively limited persistent gait improvement in term of walking speed. Results of another RCT suggested 26-week provision of passive AFO (similar to SCAR) did not have any effects on kinematic gait parameters of sub-acute stroke subjects (n = 26) [17]. More clinical trials and follow-up studies are required to generalize these results.

There were limitations in the current study. First, the sample size was relatively small for an RCT study. As discussed in the effect size analysis, a larger sample size might provide more statistically significant effects. Second, most stroke subjects still could not achieve independent stair ambulation without the robot assistance, even after the robot-assisted stair training. Stair ambulation recording and comprehensive gait analysis were not available due to the safety concern when the sub-acute subjects need to transfer from the hospital to the gait analysis laboratory with stairs and harness system. Third, the study design has not strictly controlled the training intensity and content of the routine conventional training prescribed by therapists outside the robot-assisted training session. This might cause potential bias in the CT group, who only received conventional training. Lastly, the 0.5 kg device weight on the affected side could influence the gait pattern of stroke subjects, especially the device weight was not balanced on the unaffected side. More lightweight design should be considered in the future study. Future study could also investigate the effects of robot-assisted training for overground walking with or without stair ambulation training.

## Conclusion

In summary, this two-center RCT showed the efficacy of 20-session robot-assisted training on sub-acute stroke survivors with stair and over-ground walking. The power-assisted ankle robot had better functional improvement in gait independency and walking speed than conventional training. The active powered ankle assistance might play an important role to facilitate subjects to walk more and faster with their paretic leg during stair and over-ground walking. We speculated that the incorporation of rehabilitation ankle robotics in intensive stair training could be considered in clinical rehabilitation protocol to further enhance gait recovery of stroke survivors, as well as ameliorate the workload of therapists.

## Data Availability

All data generated or analyzed during this study are included within the article. Information on this clinical trial (Clinical Trial Identifier: NCT03184259) can be found at: https://clinicaltrials.gov/ct2/show/NCT03184259.

## Abbreviations

AFO: Ankle–foot orthosis
ANCOVA: Analysis of covariance
BBS: Berg balance scale
CT: Conventional training
FAC: Functional ambulatory category
MAS: Modified Ashworth Scale
MCID: Minimal clinically important difference
PAAR: Power-assisted ankle robot
RCT: Randomized controlled trial
SCAR: Swing-controlled ankle robot
10MWT: Timed 10-m walk test
